# Improved liver fat and $R_{2}^{*}$ quantification at 0.55 T using locally low‐rank denoising

**DOI:** 10.1002/mrm.30324

**Published:** 2024-10-09

**Authors:** Shu‐Fu Shih, Bilal Tasdelen, Ecrin Yagiz, Zhaohuan Zhang, Xiaodong Zhong, Sophia X. Cui, Krishna S. Nayak, Holden H. Wu

**Affiliations:** ^1^ Department of Radiological Sciences University of California Los Angeles Los Angeles California USA; ^2^ Department of Bioengineering University of California Los Angeles Los Angeles California USA; ^3^ Ming Hsieh Department of Electrical and Computer Engineering University of Southern California Los Angeles California USA; ^4^ MR R&D Collaborations Siemens Medical Solutions USA, Inc. Los Angeles California USA

**Keywords:** 0.55 T MRI, denoising, liver PDFF, liver $R_{2}^{*}$, locally low‐rank

## Abstract

**Purpose:**

To improve liver proton density fat fraction (PDFF) and $R_{2}^{*}$ quantification at 0.55 T by systematically validating the acquisition parameter choices and investigating the performance of locally low‐rank denoising methods.

**Methods:**

A Monte Carlo simulation was conducted to design a protocol for PDFF and $R_{2}^{*}$ mapping at 0.55 T. Using this proposed protocol, we investigated the performance of robust locally low‐rank (RLLR) and random matrix theory (RMT) denoising. In a reference phantom, we assessed quantification accuracy (concordance correlation coefficient [$\rho_{c}$] vs. reference values) and precision (using SD) across scan repetitions. We performed in vivo liver scans (11 subjects) and used regions of interest to compare means and SDs of PDFF and $R_{2}^{*}$ measurements. Kruskal–Wallis and Wilcoxon signed‐rank tests were performed (*p* < 0.05 considered significant).

**Results:**

In the phantom, RLLR and RMT denoising improved accuracy in PDFF and $R_{2}^{*}$ with $\rho_{c}$ >0.992 and improved precision with >67% decrease in SD across 50 scan repetitions versus conventional reconstruction (i.e., no denoising). For in vivo liver scans, the mean PDFF and mean $R_{2}^{*}$ were not significantly different between the three methods (conventional reconstruction; RLLR and RMT denoising). Without denoising, the SDs of PDFF and $R_{2}^{*}$ were 8.80% and 14.17 s^−1^. RLLR denoising significantly reduced the values to 1.79% and 5.31 s^−1^ (*p* < 0.001); RMT denoising significantly reduced the values to 2.00% and 4.81 s^−1^ (*p* < 0.001).

**Conclusion:**

We validated an acquisition protocol for improved PDFF and $R_{2}^{*}$ quantification at 0.55 T. Both RLLR and RMT denoising improved the accuracy and precision of PDFF and $R_{2}^{*}$ measurements.

## 1 INTRODUCTION

Proton density fat fraction (PDFF)^1^ and $R_{2}^{*}$ ^2^ are powerful noninvasive MRI biomarkers for liver fat and iron accumulation, respectively. These two parameters can be quantified simultaneously using multi‐echo gradient‐echo Dixon MRI sequences followed by signal fitting to a model that resolves different confounding factors.^3^, ^4^, ^5^ Several MRI sequences and signal fitting approaches have been developed and validated at 1.5 and 3 T.^3^, ^4^, ^5^, ^6^, ^7^, ^8^, ^9^, ^10^ Recently, MRI field strengths <1.5 T are being explored due to advantages such as reduced hardware and siting costs and reduction of artifacts in certain applications.^11^, ^12^, ^13^, ^14^ A lower‐field MRI system with a larger bore diameter may also improve comfort^15^ for populations with obesity and at risk for fatty liver disease. In addition, decreased $R_{2}^{*}$ at lower fields can enable more accurate $R_{2}^{*}$ quantification in patients with high iron overload.^16^

Most existing scan protocols for joint PDFF and $R_{2}^{*}$ quantification have been designed and validated at 1.5 and/or 3 T. Adaptation to lower field strengths such as 0.55 T requires careful investigation into the trade‐offs associated with acquisition parameter choices. There are several important considerations. First, lower B_0_ field strengths result in lower equilibrium polarization, which reduces the SNR.^11^, ^12^, ^13^, ^14^ This is exacerbated when a small flip angle (FA) is used to reduce T_1_‐related bias in PDFF quantification.^17^ Low SNR can degrade image quality and affect accuracy and precision of quantitative biomarkers.^18^, ^19^, ^20^ Second, the smaller fat–water frequency difference at lower fields results in longer out‐of‐phase (TE_op_ = 6.47 ms) and in‐phase (TE_in_ = 12.94 ms) TEs. This increases scan time and limits sequence parameter choices. Increasing the number of scan repetitions to improve SNR, a common strategy, may be infeasible in breath‐holding abdominal scans. Compromises in imaging parameters such as reducing image resolution and restricting volumetric coverage can reduce diagnostic quality.

Locally low‐rank principal component analysis (PCA)–based denoising is one popular approach to suppress noise in multi‐contrast MR images. By suppressing principal components associated with smaller coefficients, noise can be reduced, whereas signal can be largely preserved. Difficulties in this type of method involve how to accurately estimate the signal rank and suppress the noise without removing the desired signal. Different approaches have been proposed to objectively estimate the noise level for effective noise suppression. One method, termed as *robust locally low‐rank denoising* (RLLR) technique,^21^ has been proposed. Using samples of random matrices from a known Gaussian distribution, the noise level in the multi‐echo images can be estimated. Based on Stein's unbiased risk estimate,^22^, ^23^ the singular value threshold can be objectively obtained for noise suppression. RLLR has been shown to improve image quality for PDFF and $R_{2}^{*}$ quantification at 3 T,^24^ but it has not been studied at lower field strengths. On the other hand, *random matrix theory* (RMT)–based denoising^25^, ^26^, ^27^ can accurately estimate noise level and remove the noise components by leveraging the spectral properties of random Gaussian matrices predicted by the Marchenko–Pastur Law.^28^ This approach has shown promising noise suppression results, especially in diffusion MRI where many contrasts (i.e., multiple *b*‐values and multiple directions) are available to construct locally low‐rank patches.^25^, ^26^, ^27^, ^29^, ^30^, ^31^ There are initial studies applying RMT‐based denoising for lower‐field MRI,^31^ but this has not yet been well studied for the application of PDFF and $R_{2}^{*}$ mapping.

In this study, our objective is to improve liver PDFF and $R_{2}^{*}$ quantification accuracy and precision at 0.55 T by (1) systematically refining and validating the acquisition parameter choices, and (2) investigating the performance of two locally low‐rank PCA‐based denoising methods: RLLR and RMT denoising. First, we performed a Monte Carlo simulation to investigate the impact of acquisition parameter choices on the accuracy and precision of PDFF and $R_{2}^{*}$ mapping at 0.55 T. Second, we used the proposed acquisition protocol informed by simulation results and performed experiments in a reference phantom. Third, we evaluated the denoising performance in the pelvis, where high‐SNR reference images can be obtained without breath‐hold limitations. Fourth, we conducted experiments with breath‐holding liver scans and compared the performance of PDFF and $R_{2}^{*}$ quantification with different reconstruction/denoising algorithms.

## 2 METHODS

### 2.1 Acquisition protocol for PDFF and $R_{2}^{*}$ quantification at 0.55 T

The choice of TEs and FA in the 3D multi‐echo gradient‐echo Dixon sequence affects PDFF and $R_{2}^{*}$ quantification accuracy.^32^, ^33^, ^34^ A common choice at 3 T is six echoes at either out‐of‐phase or in‐phase TEs and a low FA of 3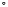 to 5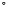 for reducing the T_1_‐related bias in PDFF estimation.^5^ Due to the longer out‐of‐phase and in‐phase TEs at 0.55 T, this strategy would lead to longer TEs and TR that prolong acquisition beyond the acceptable time for one breath‐hold. On the other hand, the T_1_‐related bias is reduced at 0.55 T because of the shortened T_1_ values and the increased TR. A larger FA that balances between SNR and the T_1_‐related bias may be considered. Because the $R_{2}^{*}$ values change with the field strength,^16^ TEs for accurate $R_{2}^{*}$ quantification should also be reconsidered. Therefore, we conducted a Monte Carlo simulation to investigate different choices of FA, the first TE, and the echo spacing (∆TE) with a range of reference PDFF and $R_{2}^{*}$ values at 0.55 T. We limited our simulation to six echoes. This consideration was to maintain a balance between sufficient number of echoes for quantification and reasonable scan time.

The signal $s\left(t_{m}\right)$ at the *m*‐th TE was simulated using the signal model:

(1) $$\begin{array}{ll} s\left(t_{m}\right) & =M\left(\left(1-F\right)+F\times \left(\sum_{j=1}^{7}a_{j}\times e^{i2\pi f_{j}t_{m}}\right)\right)\\ & \;\times e^{-R_{2}^{*}t_{m}}\times e^{-i2{\pi \mathrm{\varphi t}}_{m}}+n, \end{array}$$

where M represents the steady‐state magnetization signal dependent on the TE, TR, T_1_, and FA; F represents the PDFF value; $a_{j}$ and $f_{j}$ represent the relative amplitudes and frequencies for a seven‐peak fat spectrum^35^; φ represents the frequency shift due to B_0_ field inhomogeneity; and n represents the complex‐valued Gaussian noise.

We used T_1_ of 339 and 187 ms for water and fat protons in the liver, respectively, based on previous work that measured in vivo relaxation times at 0.55 T.^36^ The simulated FA were in the range of 2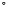 to 20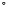. The simulated first TEs and ∆TE were both in the range of 1.2 to 2.8 ms, considering hardware specifications of the 0.55 T scanner and reasonable acquisition time of one breath‐hold. The TR was set to include all the echoes and the spoiler gradient. When investigating PDFF accuracy and precision in the range of 0% to 40% (a range that covers most of the biopsy‐proven metabolic dysfunction–associated steatotic liver disease patients with histologic steatosis grade 0 to 3^37^), the reference $R_{2}^{*}$ value was fixed at 30 s^−1^ ($R_{2}^{*}$ value at 0.55 T with no iron overload^16^). When investigating $R_{2}^{*}$ accuracy and precision in the range of 20 to 90 s^−1^ (a range that covers mild, moderate, and no iron overload at 0.55 T^16^), the reference PDFF value was fixed at 5% (close to the common cutoff value for metabolic dysfunction–associated steatotic liver disease diagnosis^38^).

For each combination of parameters (FA, first TE, ∆TE, reference PDFF, and reference $R_{2}^{*}$), 500 simulated instances were generated. For each instance, φ was randomly drawn from a range of (−100, 100) Hz. The complex‐valued noise was modeled as $n=n_{r}+i\cdot n_{i}$, where $n_{r}$ and $n_{i}$ were independently drawn from a Gaussian distribution with the same variance $\sigma^{2}$. The value of $\sigma^{2}$ was set to be similar to the noise level in actual in vivo liver scans at 0.55 T. To be more specific, the resulting apparent SNR (aSNR), defined as signal mean divided by noise SD, equaled 10 when PDFF = 5%, $R_{2}^{*}$ = 25 s^−1^, and flip angle = 8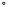 in our Monte Carlo simulation.

All the simulated instances were fitted to a seven‐peak fat model^35^ with a single $R_{2}^{*}$ decay term using a multi‐step adaptive approach.^5^ We measured the quantification accuracy by reporting the mean difference (MD) across instances of fitted PDFF and $R_{2}^{*}$ versus the reference values (i.e., the bias) at different parameter settings. We measured the quantification precision by reporting the SD across instances of fitted PDFF and $R_{2}^{*}$ at different parameter settings.

### 2.2 Locally low‐rank PCA‐based denoising

**FIGURE 1:**
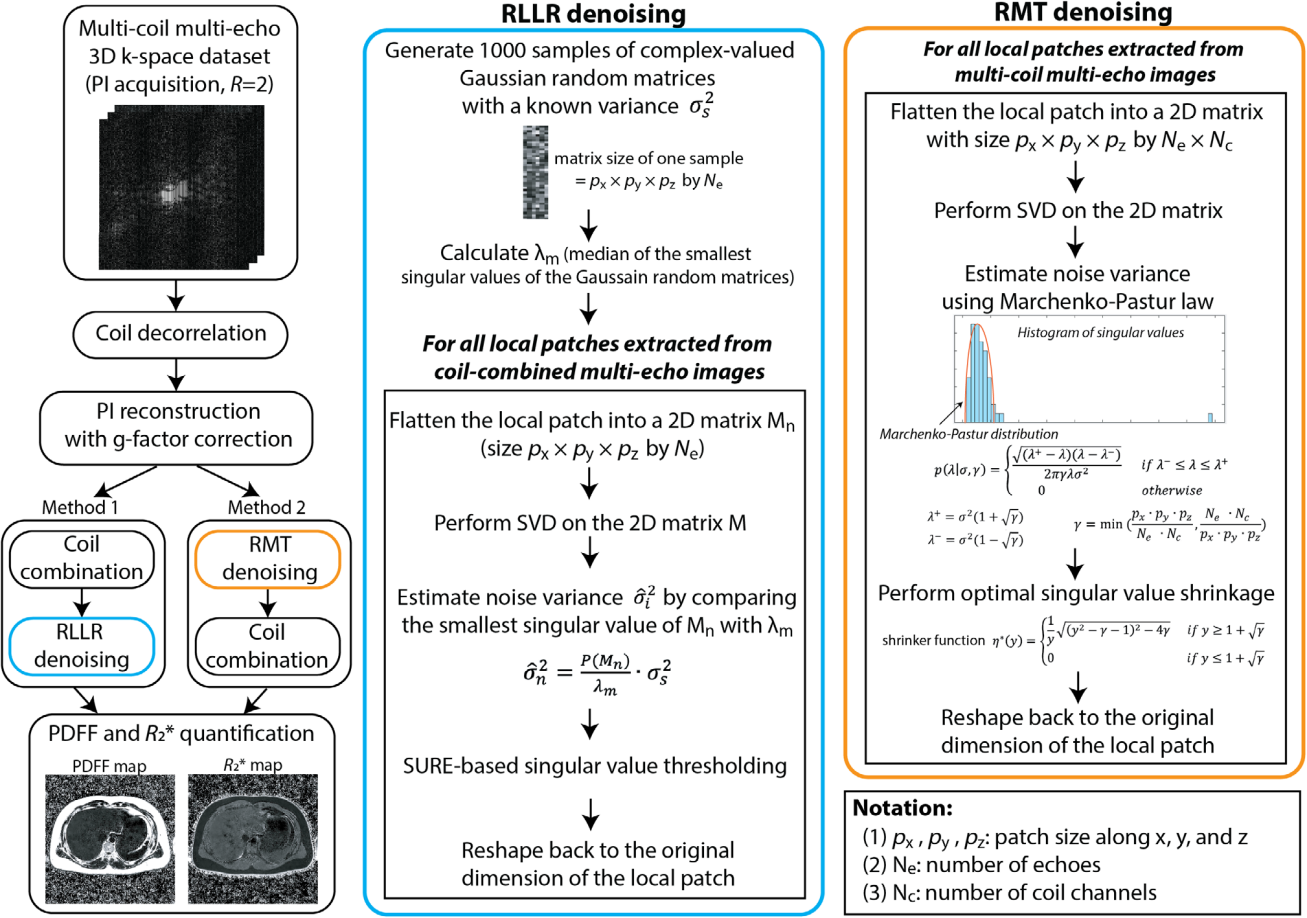
Reconstruction pipelines of the two locally low‐rank principal component analysis–based denoising methods used in this work. RLLR denoising was applied on coil‐combined multi‐contrast images, whereas RMT denoising was applied on the multi‐coil multi‐contrast images. Both RLLR and RMT denoising methods needed to accurately estimate noise variance before performing singular value thresholding or shrinkage to suppress Gaussian noise. PDFF, proton density fat fraction; RLLR, robust locally low‐rank; RMT, random matrix theory; SVD, singular value decomposition; SURE, Stein's unbiased risk estimate.

Here, we briefly summarize the two techniques that were investigated in this work, RLLR and RMT denoising (Figure 1), and describe how we adapt them to our specific application. More technical details can be found in previous works.^21^, ^25^ In the following paragraphs, we use *p*_x_, *p*_y_, and *p*_z_ to represent the patch size in the three image dimensions, and use *N*_e_ and *N*_c_ to represent the number of echoes and number of coil channels, respectively.

The RLLR denoising method constructs a 2D low‐rank complex valued matrix $M_{n}$ with dimensions [*p*_x_ × *p*_y_ × *p*_z_ by *N*_e_] from the coil‐combined multi‐echo images. Assuming the signal rank of $M_{n}$ is smaller than *N*_e_, the component associated with the smallest singular value is mainly noise. Before noise reduction, 2D random Gaussian matrix samples with dimensions [*p*_x_ × *p*_y_ × *p*_z_ by *N*_e_] were generated using a predetermined variance $\sigma_{s}^{2}$. The median of the smallest singular values of these matrix samples, denoted as $\lambda_{m}$, is calculated. By comparing the smallest singular value of $M_{n}$ to $\lambda_{m}$, the noise variance ${\hat{\sigma }}_{n}^{2}$ can be estimated using

(2) $${\hat{\sigma }}_{n}^{2}=\frac{P\left(M_{n}\right)}{\lambda_{m}}\times \sigma_{s}^{2},$$

while P(⋅) extracts the smallest singular values of its argument. After estimating ${\hat{\sigma }}_{n}^{2}$, RLLR denoising finds the optimal value for singular value soft‐thresholding by minimizing Stein's unbiased risk estimate^23^ and obtaining the denoised matrix. All the overlapping local patches (with stride = 1 along three spatial dimensions) are denoised using the same method and averaged to generate the final denoised images. Please note that previous works applied RLLR denoising on PDFF and $R_{2}^{*}$ mapping at 3 T and only used a 2D low‐rank matrix constructed from two image dimensions.^21^, ^24^ In this work, we extended the method to include the slice dimension.

The RMT denoising method relies on the Marchenko–Pastur law.^28^ Let us consider a 2D random matrix X with dimensions [p by q] (p≤q) whose entries are drawn from a Gaussian distribution of mean 0 and variance $\sigma^{2}$. The probability density function of the eigenvalues λ of the matrix $Y=\frac{1}{q}XX^{T}$ can be described by the Marchenko–Pastur distribution:

(3) $$p\left(\lambda |\sigma^{2},\gamma \right)=\left\{\begin{array}{ll} \frac{\sqrt{\left(\lambda^{+}-\lambda \right)\left(\lambda -\lambda^{-}\right)}}{2\pi \gamma \lambda \sigma^{2}} & \text{if}\;\lambda^{-}\leq \lambda \leq \lambda^{+}\\ 0 & \text{otherwise} \end{array}\right.,$$

where $\lambda^{+}=\sigma^{2}\left(1+\sqrt{\gamma }\right)$, $\lambda^{-}=\sigma^{2}\left(1-\sqrt{\gamma }\right)$, γ=p/q. After constructing a low‐rank matrix from local image patches, noise variance $\sigma^{2}$ can be estimated by comparing the distribution of the singular values of the low‐rank matrix to the Marchenko–Pastur distribution. Because it requires a sufficient number of eigenvalues/singular values for accurate estimation of the noise variance, we use both echo and coil dimensions to construct low‐rank matrices. The real and imaginary components from the multi‐coil multi‐echo complex data are then concatenated to form a matrix with dimensions [*p*_x_ × *p*_y_ × *p*_z_ by 2 × *N*_e_ × *N*_c_]. Once the noise variance is estimated, optimal singular value shrinkage based on Frobenius norm minimization^39^ is used. All the overlapping local patches are denoised using the same method and averaged to generate the denoised multi‐coil multi‐echo images. Coil combination^40^ is performed after RMT denoising.

Both denoising methods assume the noise is Gaussian‐distributed. Therefore, the reconstruction pipeline includes coil decorrelation^41^ and requires g‐factor correction^42^ for parallel imaging (PI)‐accelerated data before denoising (Figure 1).

### 2.3 PDFF and $R_{2}^{*}$ phantom imaging

We validated the PDFF and $R_{2}^{*}$ quantification accuracy using a reference phantom (Calimetrix, Madison, WI) with seven PDFF‐only vials (0% to 100%, reference values provided by the vendor) and 10 $R_{2}^{*}$‐only vials (17.7 to 1009.5 s^−1^ measured at 1.5 T, provided by the vendor). The PDFF‐only vials did not have controlled $R_{2}^{*}$ values, and the $R_{2}^{*}$‐only vials had no fat content. Scans were performed using a whole‐body 0.55 T MRI system (prototype MAGNETOM Aera, Siemens Healthineers, Erlangen, Germany) equipped with high‐performance shielded gradients (45 mT/m maximum amplitude, 200 T/m/s slew rate). Phased‐array receiver coils (18‐channel spine array and six‐channel body array) were used, and there were *N*_c_ = 12 activated coil channels during the scans. To acquire phantom images with similar SNR as in the in vivo liver scans, we placed pads between phantom vials and the coils such that the space between the body array coil and the spine array coil was similar to the volume of an adult abdomen. We acquired data using a 3D multi‐echo gradient‐echo Dixon MRI research application sequence.^5^ Key sequence parameters, based on findings from our Monte Carlo simulation, included *N*_e_ = 6 with TEs = (2.16, 4.32, 6.48, 8.64, 10.8, 12.96) ms, TR = 14.7 ms, FA = 8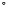, FOV = 300 × 300 mm^2^, matrix size = 192 × 192, and slice thickness = 5 mm. PI with acceleration factor (*R*) of 2 was used. The scan was repeated 50 times without repositioning. Detailed sequence parameters are reported in Table 1. Each scan repetition was reconstructed individually, using three reconstruction methods: (1) conventional reconstruction with only PI reconstruction (GRAPPA) without any denoising, (2) PI reconstruction and RLLR denoising with an image patch size (*p*_x_, *p*_y_, *p*_z_) = (5, 5, 5), and (3) PI reconstruction and RMT denoising with an image patch size (5, 5, 5). The reconstructed images were fitted with a multi‐step adaptive approach^5^ accounting for fat model complexity^35^ and single $R_{2}^{*}$ decay to generate PDFF and $R_{2}^{*}$ maps. For a fair comparison, all the reconstruction results presented in this work were reconstructed offline with the same GRAPPA algorithm and the same fat–water‐$R_{2}^{*}$ fitting method.

**TABLE 1:** Sequence parameters for phantom, in vivo pelvis, and in vivo liver MRI scans at 0.55 T.

	Phantom		In vivo pelvis	In vivo liver
	2D multi‐echo gradient echo	3D multi‐echo Dixon	3D multi‐echo Dixon	3D multi‐echo Dixon
Acquisition orientation	Axial	Axial	Axial	Axial
FOV (mm × mm)	300 × 300	300 × 300	400 × 400	380 × 380
TE (ms)	1.35, 3.5, 5.8, 8.0, 10.3, 12.6, 14.8, 17.1, 19.3, 21.6, 23.9, 26.1	2.16, 4.32, 6.48 (OP), 8.64, 10.8, 12.96 (IP)	2.16, 4.32, 6.48 (OP), 8.64, 10.8, 12.96 (IP)	2.16, 4.32, 6.48 (OP), 8.64, 10.8, 12.96 (IP)
TR (ms)	35	14.7	14.7	14.7
Matrix size	160 × 160	192 × 192	192 × 192	192 × 192
In‐plane resolution (mm × mm)	1.9 × 1.9	1.6 × 1.6	2.1 × 2.1	2.0 × 2.0
Number of slices	1	8	8	8
Slice oversampling	N/A	20%	20%	20%
Slice thickness (mm)	5	5	5	5
Flip angle ( 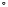 )	15	8	8	8
Bandwidth (Hz/px)	1565	590	590	590
Parallel imaging	No	GRAPPA (*R* = 2)	GRAPPA (*R* = 2)	GRAPPA (*R* = 2)
Averages	2	1	1	1
Scan time (min:s)	0:12	0:19	0:19	0:19 (breath‐hold)

N/A, not applicable. OP, out of phase. IP, in phase.

The analysis consisted of two parts. First, we assessed the agreement of PDFF and $R_{2}^{*}$ values from different reconstruction methods versus the reference for the evaluation of accuracy. In this part, results from one scan repetition were used. We placed a region of interest (ROI) in each vial and calculated the mean PDFF and $R_{2}^{*}$. PDFF values provided by the phantom vendor were used as the reference. To obtain the reference $R_{2}^{*}$ values at 0.55 T, a single‐slice 12‐echo gradient‐echo sequence was scanned and the images were fitted to a mono‐exponential model.^16^ Two $R_{2}^{*}$ vials had $R_{2}^{*}$ > 250 s^−1^ ($T_{2}^{*}$ < 4 ms) at 0.55 T, which could not be reliably fitted using the specified protocol and were not included in the quantitative analysis. The phantom vials analyzed spanned the entire PDFF range (0% to 100%) and contained $R_{2}^{*}$ values that are consistent with values reported in subjects without iron overload ($R_{2}^{*}$ < 45 s^−1^ from previous work^16^) and with mild liver iron overload (45 s^−1^ < $R_{2}^{*}$ < 91 s^−1^ from previous work^16^). The MD and the concordance correlation coefficient ($\rho_{c}$)^43^ between the measured PDFF and $R_{2}^{*}$ values versus the reference were calculated to assess agreement. Linear regression was also performed. Second, we evaluated the precision by calculating the SD of quantitative measurements in each voxel across scan repetitions. The mean values of the change in PDFF and $R_{2}^{*}$ SDs between different reconstruction methods were reported.

### 2.4 In vivo pelvic imaging

Quantitatively assessing denoising performance in liver scans can be challenging due to the difficulty to obtain reference high‐SNR images from multiple scan repetitions. The liver position can vary across multiple breath‐holds, leading to artifacts after averaging. Therefore, we performed an experiment in the pelvis to quantify accuracy and precision of in vivo PDFF and $R_{2}^{*}$ mapping. The experiment contained two analyses: (1) to investigate the denoising performance and the quantification accuracy under different noise levels, and (2) to investigate the quantification precision by calculating the SDs of PDFF and $R_{2}^{*}$ measurements across scan repetitions. All in vivo experiments in this work were conducted under a Health Insurance Portability and Accountability Act–compliant study protocol approved by the institutional review board. All subjects were scanned after providing written informed consent.

For the first analysis, we scanned a healthy volunteer (29‐year‐old male with body‐mass index [BMI] 26.4 kg/m^2^) using the 3D multi‐echo gradient‐echo Dixon MRI research application sequence^5^ with 30 scan repetitions (no repositioning). Key parameters were the same as the phantom scans except for the FOV and the in‐plane resolution. We averaged the multi‐coil multi‐echo k‐space data across the 30 repetitions to generate the “reference” k‐space data. We then added complex‐valued random Gaussian noise with different variances to the reference k‐space data to generate synthetic pelvis datasets with different noise levels. We chose the noise variances so that the synthetic images after GRAPPA reconstruction (without any denoising) had aSNR ranging from 3 to 15 (whereas the original reference image had aSNR = 95). Here, aSNR was measured by the signal mean in a muscle ROI divided by background noise SD in coil‐combined echo 3 (out‐of‐phase) images. We performed RLLR and RMT denoising on the synthetic images after GRAPPA reconstruction. PDFF and $R_{2}^{*}$ maps were reconstructed using the same signal fitting method described earlier. We placed three ROIs, each with a size of 5 mm^2^, in the subcutaneous fat tissue and in the muscle. Quantification accuracy was assessed by comparing mean PDFF and $R_{2}^{*}$ in these ROIs versus the quantification results in the reference data (from 30 repetitions).

For the second analysis, we scanned three healthy volunteers (3 males, age: 29.7 ± 0.6 years, BMI: 24.5 ± 2.6 kg/m^2^) using the same sequence, each with 15 scan repetitions. Each repetition was reconstructed individually using three different methods: (1) conventional reconstruction without denoising, (2) RLLR denoising with a patch size (5, 5, 5), and (3) RMT denoising with a patch size (5, 5, 5). PDFF and $R_{2}^{*}$ maps were calculated using the same signal fitting approach. To assess precision, we calculated pixel‐wise SDs of PDFF and $R_{2}^{*}$ values across 15 scan repetitions. We further calculated the percentage of voxels, which had reduced SDs of PDFF and $R_{2}^{*}$ (meaning improved precision) in denoised results compared to conventional reconstruction results.

### 2.5 In vivo liver imaging

Eleven subjects (three females and eight males, age: 39.5 ± 14.3 years, BMI: 26.3 ± 4.0 kg/m^2^) were recruited and scanned. Four of the subjects (one female and three males, age: 49.5 ± 16.8 years, BMI: 29.9 ± 2.9 kg/m^2^) had known fatty liver. All the subjects were scanned using the 3D multi‐echo gradient‐echo Dixon research application sequences^5^ (Table 1) within a single breath‐hold. Conventional reconstruction (no denoising, only GRAPPA) and reconstruction with the two denoising methods were performed. The same signal fitting approach was used to generate PDFF and $R_{2}^{*}$ maps.

For each subject, three circular ROIs, each with a size of 5 mm^2^, were placed on three different axial slices in the liver while avoiding large vessels.^7^ Mean and SD of the PDFF and $R_{2}^{*}$ values within each ROI were recorded. Bland–Altman analysis was performed to analyze the agreement of the quantification results between the conventional reconstruction and two different denoising methods.

We performed Kruskal–Wallis tests to investigate if there were any differences in PDFF mean, $R_{2}^{*}$ mean, PDFF SD, and $R_{2}^{*}$ SD in liver ROIs among the three reconstruction methods. *p* < 0.05 was considered significant. If the Kruskal–Wallis tests indicated significant differences, additional pair‐wise Wilcoxon signed rank tests with Bonferroni correction for the *p*‐values (*p* < 0.05/3 = 0.017 considered significant) were used to evaluate if there was significant difference between a pair of two reconstruction methods. For all the statistical tests, only one liver ROI measurement in the mid‐slice from each subject was used.

## 3 RESULTS

### 3.1 Monte Carlo simulation results

**FIGURE 2:**
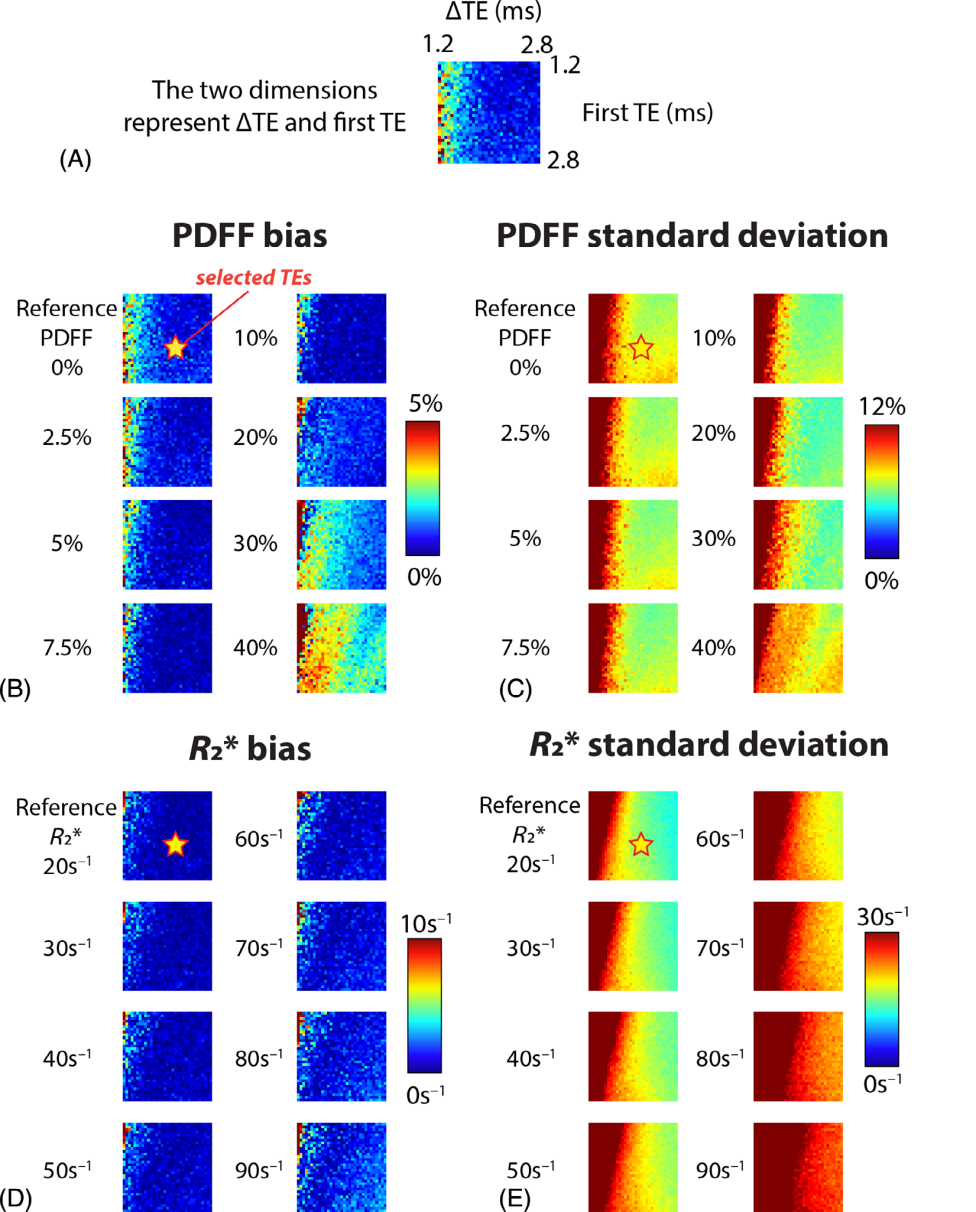
Monte Carlo simulation results of FA = 8
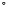
regarding the accuracy and precision for (A,B,C) PDFF and (D,E) $R_{2}^{*}$ mapping using different first TE and ∆TE at 0.55 T. Complete results from different FA can be found in Figure S1. The PDFF bias and SD were reported in absolute units. To balance between accuracy and precision of parameter quantification and breath‐holding scan time, we chose first TE = 2.16 ms and ∆TE = 2.16 ms as indicated by the stars. FA, flip angle.

The Monte Carlo simulation results from FA = 8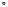 are in Figure 2 (complete results from different FA in Figure S1). A larger FA results in larger biases in PDFF due to T_1_ differences between fat and water. In contrast, a smaller FA results in less precise PDFF and $R_{2}^{*}$ due to lower SNR. Shorter TEs and less $T_{2}^{*}$ weighting in the multi‐echo signal also results in less precise PDFF and $R_{2}^{*}$. Considering the quantification accuracy and precision across a range of relevant PDFF and $R_{2}^{*}$ values, we chose FA = 8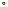, first TE = 2.16 ms, and ∆TE = 2.16 ms as the preferred setting. In this design, the third TE and the sixth TE corresponded to out‐of‐phase and in‐phase TEs at 0.55 T, respectively.

Based on the simulation results at a representative aSNR level, our selected acquisition protocol achieved PDFF biases of 0.2% to 2% and PDFF SDs of 5.4% to 7.2% for reference PDFF values ranging from 0% to 40%. The PDFF bias and SD are reported with absolute units here and throughout this work. At the same time, our selected acquisition protocol yielded $R_{2}^{*}$ biases of 0.2 to 2.2 s^−1^ and $R_{2}^{*}$ SDs of 10.5 to 17.7 s^−1^ for reference $R_{2}^{*}$ values ranging from 20 to 90 s^−1^. Please note that these simulation results did not consider any denoising.

### 3.2 PDFF and $R_{2}^{*}$ phantom imaging results

**FIGURE 3:**
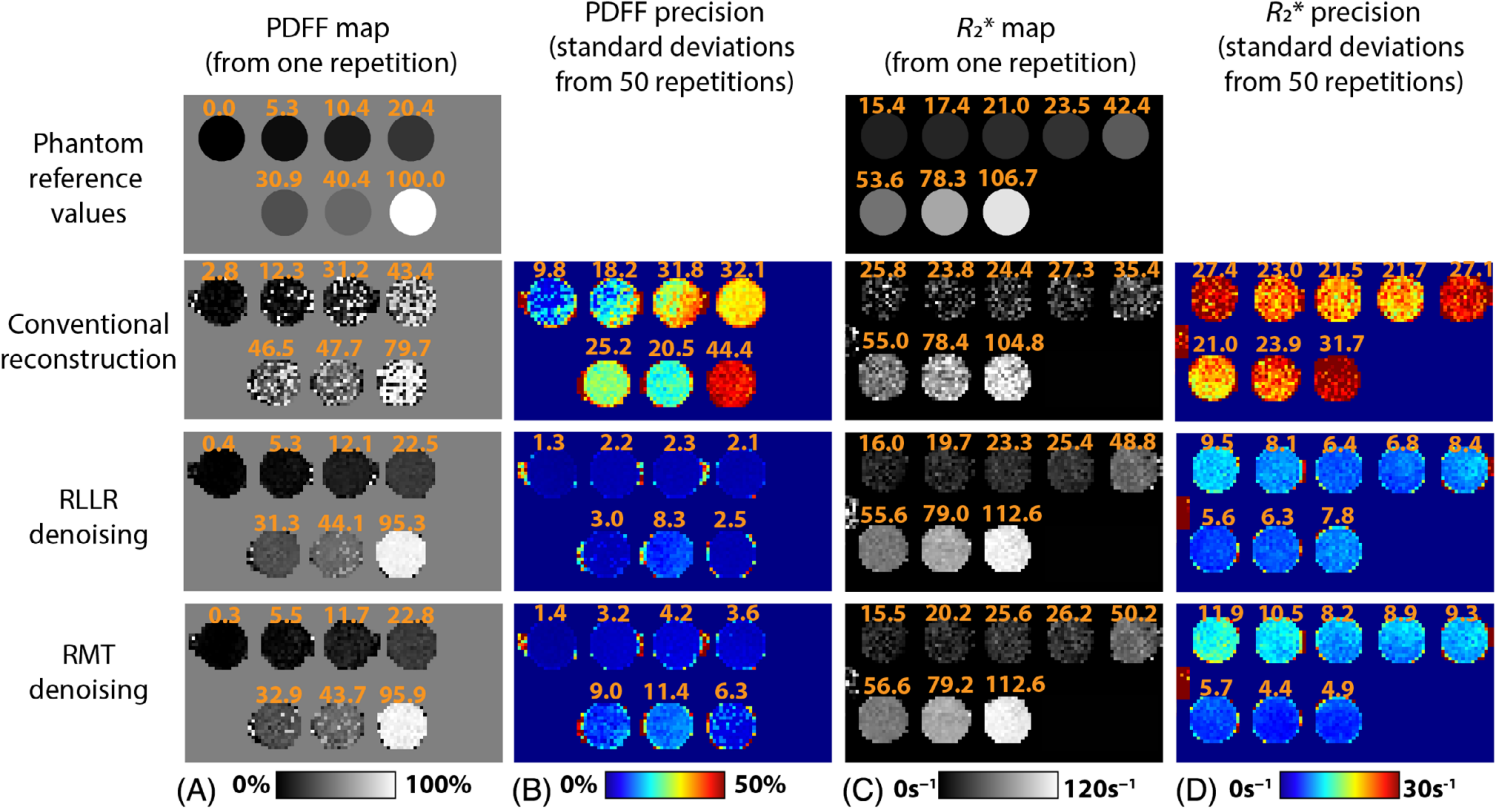
(A) PDFF quantification results in the phantom from one scan repetition. (B) Maps showing the pixelwise SD of PDFF across 50 scan repetitions. (C) $R_{2}^{*}$ quantification results in the phantom from one scan repetition. (D) Maps showing the pixelwise SD of $R_{2}^{*}$ across 50 scan repetitions. Numbers above each phantom vial show the measured mean value in that specific vial using a circular ROI. For example, the highest PDFF vial in the conventional reconstruction result had a mean PDFF of 79.7% and a SD of 44.4% in the circular ROI. ROI, region of interest.

The signal difference between denoised and non‐denoised images showed minimal structured signals, demonstrating effective noise suppression without removing desired signal (Figure S2). Figure 3A,C shows quantitative maps from one scan repetition. Without denoising, large PDFF errors and noisy PDFF and $R_{2}^{*}$ measurements were observed. Both denoising methods improved the visual quality of PDFF and $R_{2}^{*}$ maps with reduced inhomogeneity. Figure 3B,D shows maps of pixel‐wise SDs of PDFF and $R_{2}^{*}$ values from 50 scan repetitions. Compared with conventional reconstruction, RLLR denoising showed an average of 86% and 77% decrease in PDFF and $R_{2}^{*}$ SDs, respectively; RMT denoising showed an average of 77% and 67% decrease in PDFF and $R_{2}^{*}$ SDs, respectively.

**FIGURE 4:**
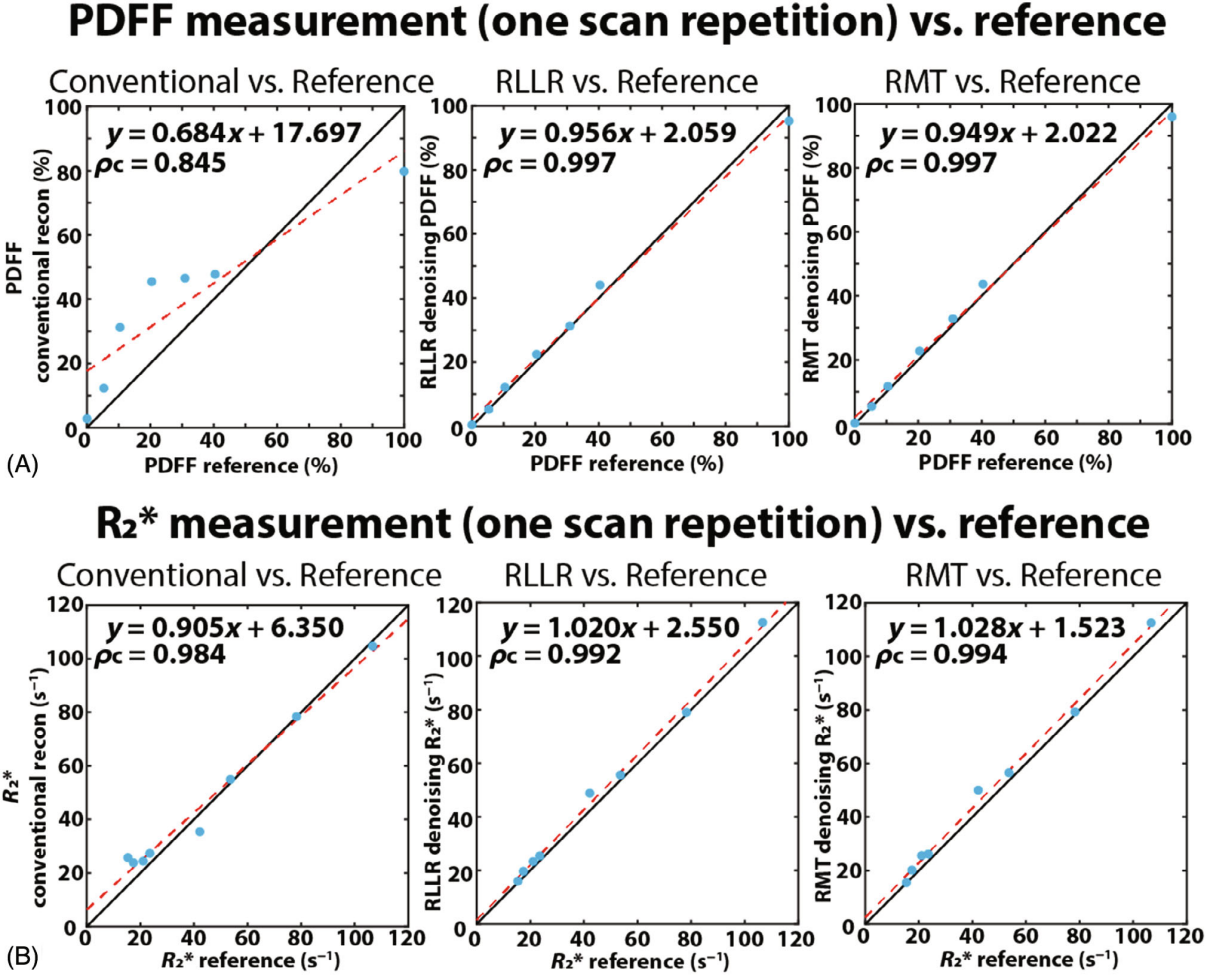
Correlation plots that compare (A) the mean PDFF measurements and (B) the mean $R_{2}^{*}$ measurements from one scan repetition with respect to the reference values. The linear regression results and the concordance correlation coefficients (*ρ*_c_) for each comparison are shown.

Compared with the reference, the MD (i.e., bias) of PDFF was 8.03% for conventional reconstruction, 0.51% for RLLR denoising, and 0.77% for RMT denoising. Compared with the reference, the MD (i.e., bias) of $R_{2}^{*}$ was 2.08 s^−1^ for conventional reconstruction, 2.76 s^−1^ for RLLR denoising, and 3.48 s^−1^ for RMT denoising. Figure 4A,B shows the correlation plots of PDFF and $R_{2}^{*}$ measurements between different methods and the reference. Conventional reconstruction had $\rho_{c}$ = 0.845 in PDFF and $\rho_{c}$ = 0.984 in $R_{2}^{*}$ when compared with the reference. Compared with reference PDFF, RLLR denoising had $\rho_{c}$ = 0.997 with regression result *y* = 0.956*x* + 2.059, and RMT denoising had $\rho_{c}$ = 0.997 with regression result *y* = 0.949*x* + 2.022. Compared with reference $R_{2}^{*}$, RLLR denoising had $\rho_{c}$ = 0.992 with regression result *y* = 1.020*x* + 2.550, and RMT denoising had $\rho_{c}$ = 0.994 with regression result *y* = 1.028*x* + 1.523. Both denoising methods achieved close PDFF and $R_{2}^{*}$ agreement with the reference.

### 3.3 In vivo pelvic imaging results

**FIGURE 5:**
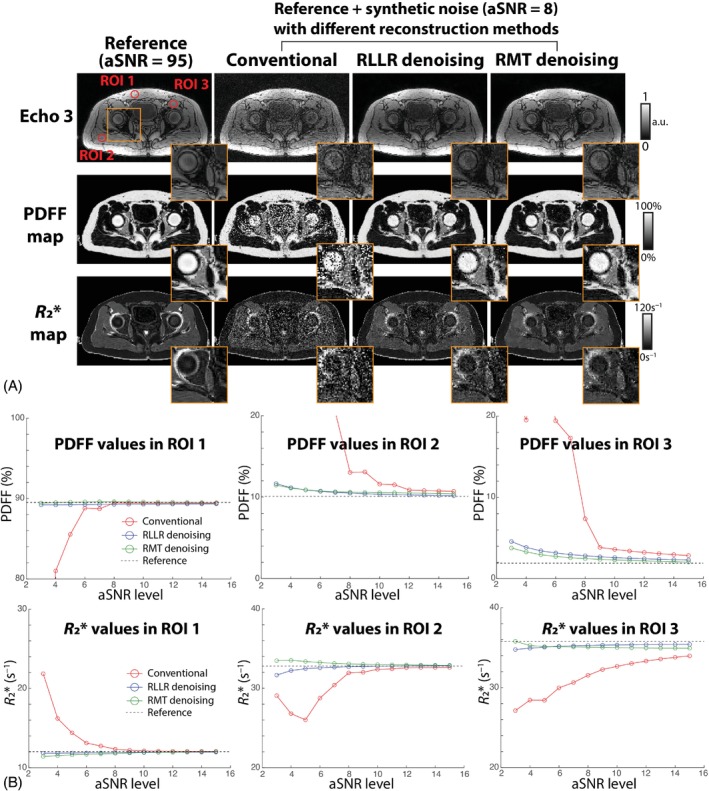
(A) Comparison of coil‐combined images and quantitative maps in the synthetic pelvis dataset (aSNR = 8) reconstructed with different methods. (B) PDFF and $R_{2}^{*}$ measurements in three ROIs (locations depicted in [A]) across different aSNR levels. Both RLLR and RMT denoising achieved better quantification accuracy (closer agreement with reference results) for PDFF and $R_{2}^{*}$ than conventional reconstruction. aSNR, apparent SNR.

Figure 5A shows the reference images (aSNR = 95) and synthetic images with aSNR = 8 reconstructed with different methods. Both denoising methods reduced PDFF quantification error and provided less noisy $R_{2}^{*}$ measurements. Without denoising, larger PDFF quantification errors were observed near the center of the body. This is consistent with the fact that the center of the body is farther away from the coil elements and the central region in the FOV has a higher g‐factor and more noise amplification.

Figure 5B compares quantification results in three ROIs across different aSNR levels. Different ROIs exhibited different levels of sensitivity to aSNR. This can be due to differences in signal intensity magnitudes and the underlying PDFF and $R_{2}^{*}$ values in different types of tissue. Both denoising methods reduced PDFF and $R_{2}^{*}$ errors. However, for images with aSNR less than 6, a PDFF bias of 1% to 2% still existed in two ROIs after denoising.

**FIGURE 6:**
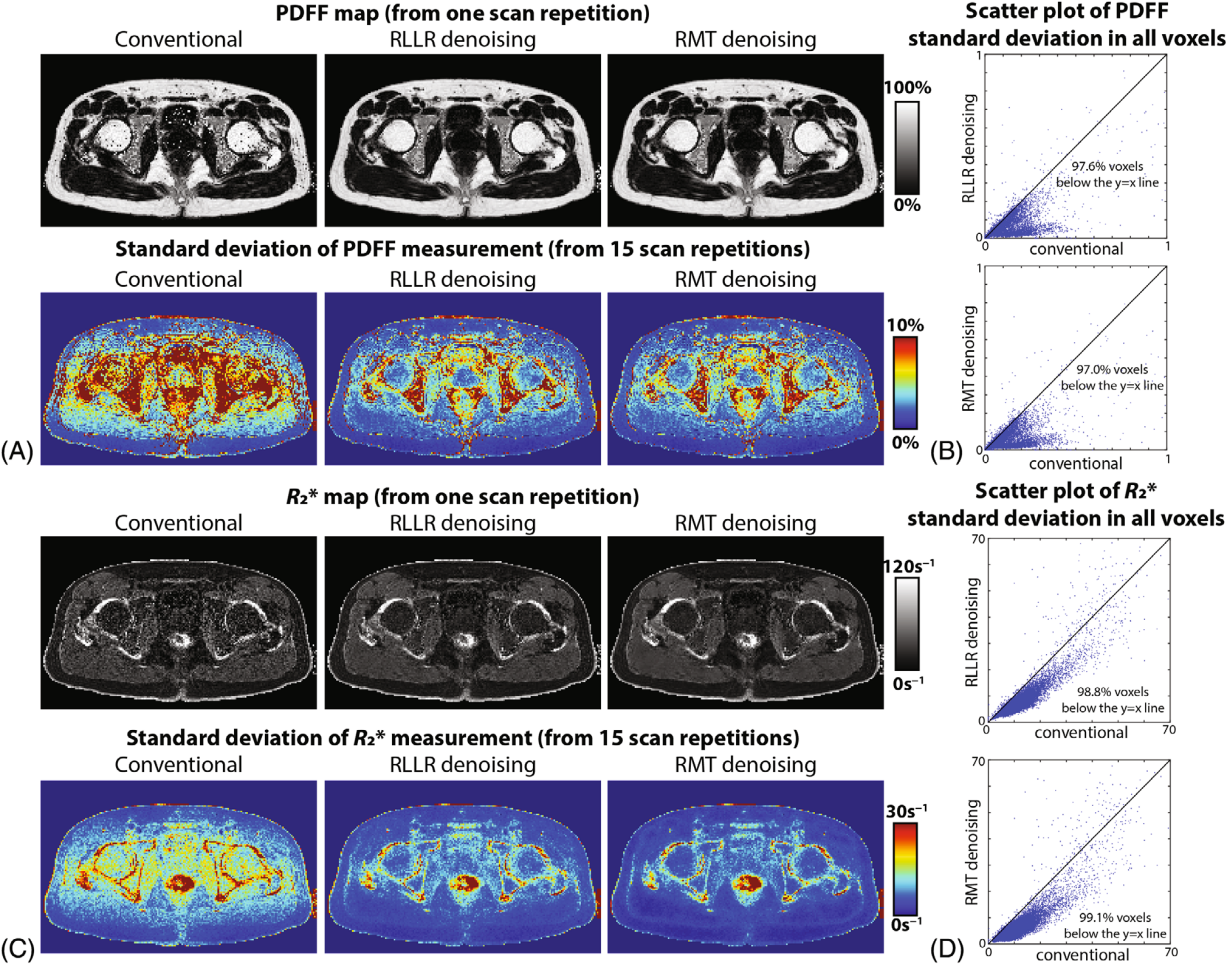
(A) Representative PDFF map and corresponding voxel‐wise PDFF SD map for different methods. (B) Scatter plot of PDFF SD in all voxels (background voxels excluded). (C) Representative $R_{2}^{*}$ map and corresponding pixel‐wise $R_{2}^{*}$ SD map for different methods. (D) Scatter plot of $R_{2}^{*}$ SD in all voxels (background voxels excluded).

Figure 6 shows representative pelvis MRI reconstruction results and SDs of PDFF and $R_{2}^{*}$ measurements across 15 scan repetitions. Compared to conventional reconstruction, both denoising methods improved PDFF and $R_{2}^{*}$ precision in terms of smaller SDs. Figure 6B,D show the scatter plots of PDFF and $R_{2}^{*}$ SDs from one representative slice. Across all the subjects in RLLR‐denoised results, the percentage of voxels with decreased PDFF and $R_{2}^{*}$ SDs were 97.5% ± 0.3% and 98.9% ± 0.4%. Across all the subjects in RMT‐denoised results, the percentage of voxels with decreased PDFF and $R_{2}^{*}$ SDs were 96.9% ± 0.4% and 98.9% ± 0.5%.

### 3.4 In vivo liver imaging results

**FIGURE 7:**
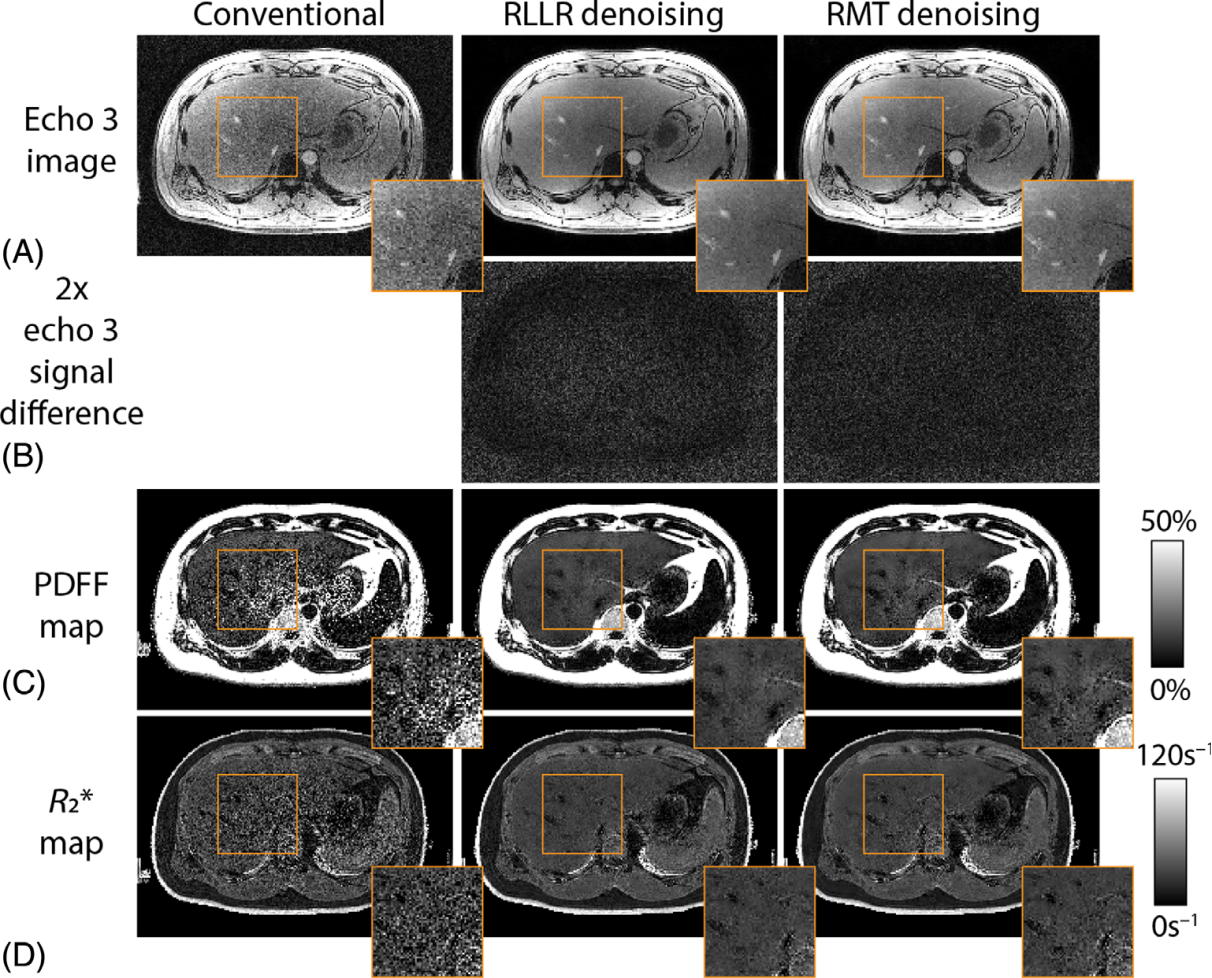
Representative result of (A) coil‐combined echo 3 out‐of‐phase image, (B) signal difference in echo 3 image, (C) PDFF map, and (D) $R_{2}^{*}$ map from a fatty liver subject (45‐year‐old male, BMI = 31.6 kg/m^2^). The signal difference between conventional reconstructed and denoised images showed minimal tissue structures, demonstrating effective noise removal. Both RLLR and RMT denoising reduced PDFF quantification errors and provided less noisy $R_{2}^{*}$ maps. BMI, body mass index.

Figure 7 shows representative results from a fatty liver subject (45‐year‐old male, BMI = 31.6 kg/m^2^). Noisy images from conventional reconstruction led to PDFF quantification error and noisy $R_{2}^{*}$ measurements. After RLLR or RMT denoising, vessels in the liver became more discernible and the PDFF and $R_{2}^{*}$ maps were less noisy.

**FIGURE 8:**
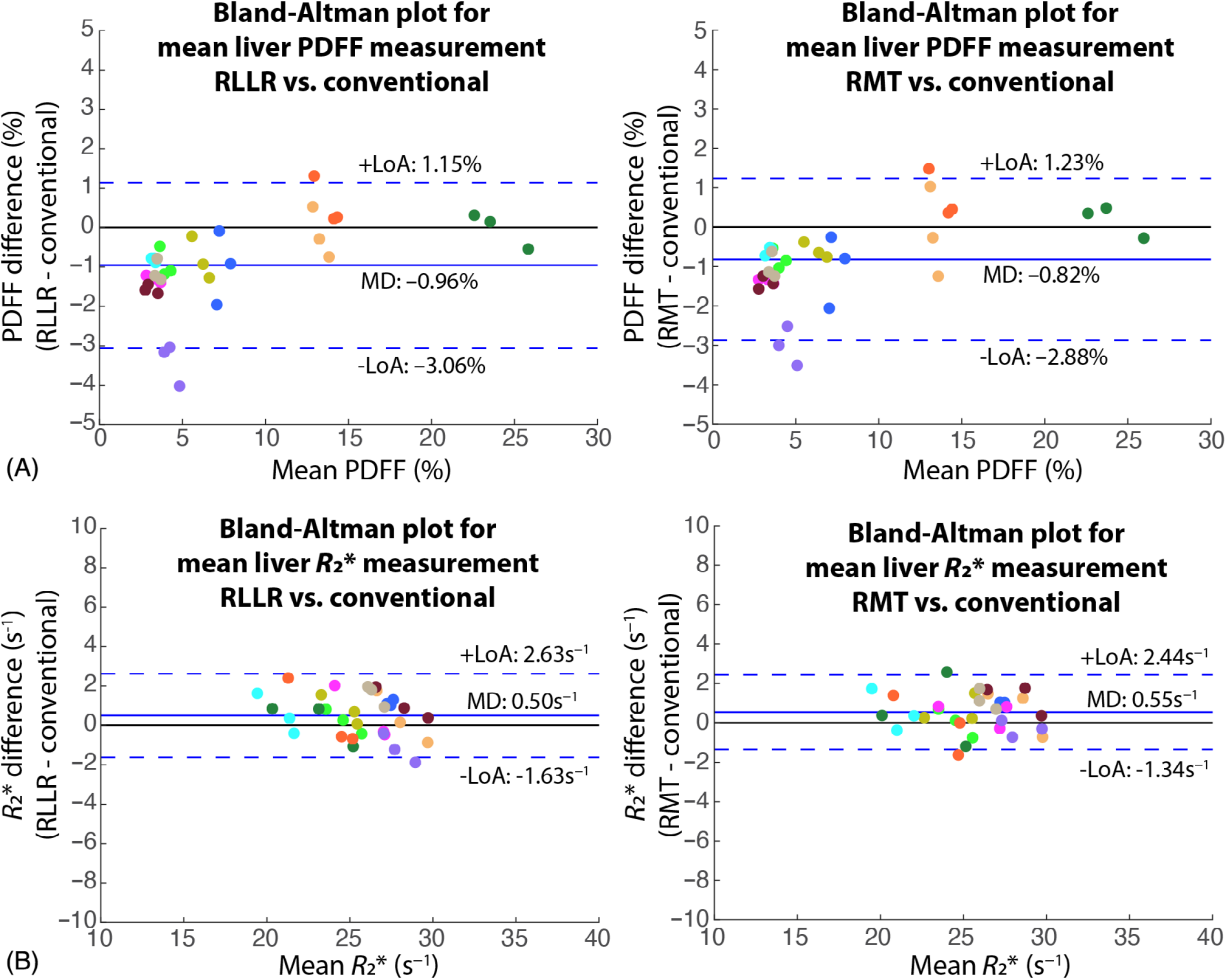
(A) Bland–Altman plots comparing mean liver PDFF measurements in results using RLLR and RMT denoising versus conventional reconstruction. (B) Bland–Altman plots comparing mean liver $R_{2}^{*}$ measurements in results using RLLR and RMT denoising versus conventional reconstruction. Three ROIs were placed in every subject, and ROIs from the same subject were color‐coded with the same color. LoA, 95% limits of agreement.

Figure 8 shows Bland–Altman plots comparing liver PDFF and $R_{2}^{*}$ values from two denoising methods versus using conventional reconstruction. For PDFF, RLLR and RMT denoising showed a MD of −0.96% and −0.82%, respectively, when compared with conventional reconstruction. This is consistent with previous findings^44^ that noise would lead to a positive PDFF bias (i.e., reducing noise can reduce the bias). On the other hand, the MD in $R_{2}^{*}$ between denoised and non‐denoised results were small, with values of 0.50 s^−1^ between RLLR denoising and conventional reconstruction and 0.55 s^−1^ between RMT denoising and conventional reconstruction.

**FIGURE 9:**
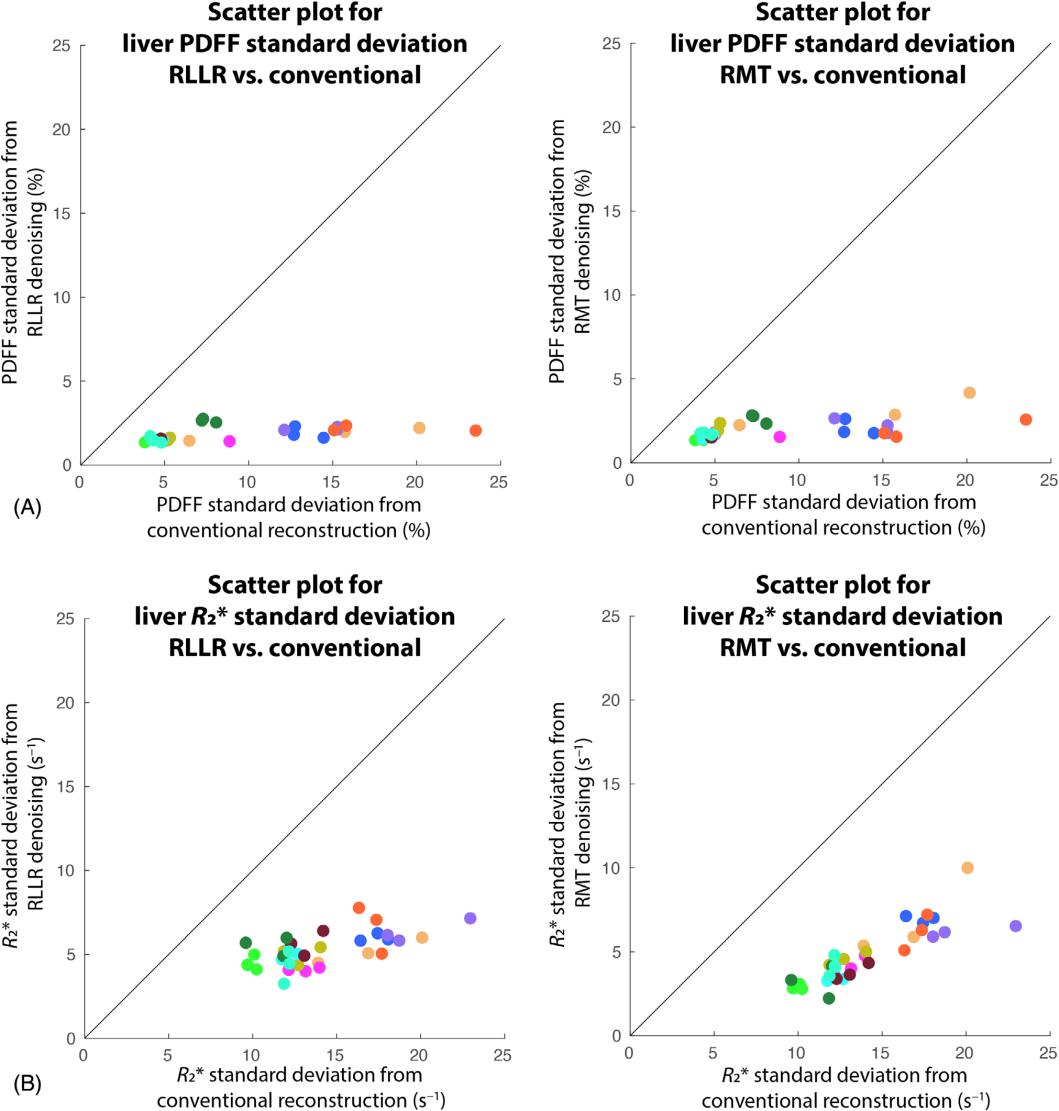
(A) Scatter plots comparing liver PDFF SD in results using RLLR and RMT denoising versus conventional reconstruction. (B) Scatter plots comparing liver $R_{2}^{*}$ SD in results using RLLR and RMT denoising versus conventional reconstruction. Both denoising methods greatly reduced SDs of PDFF and $R_{2}^{*}$ measurements in liver ROIs. Three ROIs were placed in every subject, and ROIs from the same subject were color‐coded with the same color.

Figure 9 shows scatter plots of PDFF and $R_{2}^{*}$ SDs in liver ROIs from two denoising methods versus conventional reconstruction. The mean value of PDFF SDs in liver ROIs was 8.80% for conventional reconstruction and was reduced to 1.79% and 2.00% after RLLR and RMT denoising, respectively. The mean value of $R_{2}^{*}$ SDs in liver ROIs was 14.17 s^−1^ for conventional reconstruction, and was reduced to 5.31 and 4.81 s^−1^ after RLLR and RMT denoising, respectively.

The Kruskal–Wallis (*p* < 0.05 considered significant) tests did not indicate significant differences in mean PDFF (*p* = 0.209) and mean $R_{2}^{*}$ (*p* = 0.846) among three reconstruction methods. On the other hand, the Kruskal–Wallis tests found significant differences in PDFF SDs (*p* < 0.001) and $R_{2}^{*}$ SDs (*p* < 0.001) among three reconstruction methods. In pair‐wise Wilcoxon tests, both RLLR denoising and RMT denoising had significant differences in PDFF SDs and $R_{2}^{*}$ SDs when compared with conventional reconstruction (*p* < 0.001 for all comparisons). There was no significant difference in PDFF SDs (*p* = 0.083) and $R_{2}^{*}$ SDs (*p* = 0.577) between RLLR denoising and RMT denoising.

## 4 DISCUSSION

In this work, we refined and validated the acquisition parameter choices for PDFF and $R_{2}^{*}$ quantification at 0.55 T and investigated the performance of two denoising methods to improve the quantification accuracy and precision. Based on the Monte Carlo simulation, we designed a six‐echo protocol for quantifying liver PDFF and $R_{2}^{*}$ at 0.55 T. Even with our careful design of acquisition parameters at 0.55 T, the resulting biases and appreciable SDs of PDFF and $R_{2}^{*}$ underscored the importance and need of denoising algorithms. Using the proposed protocol in phantom and pelvis scans, we demonstrated that both RLLR and RMT denoising improved quantification accuracy in terms of close agreements with the reference and improved quantification precision in terms of reduced SDs across scan repetitions. In a cohort of 11 subjects, RLLR and RMT denoising significantly reduced SDs of PDFF and $R_{2}^{*}$ measurements in the liver ROIs when compared to conventional reconstruction.

To determine an acquisition protocol that can estimate PDFF and $R_{2}^{*}$ values in a range that is relevant for patient cohorts, we focused on PDFF values from 0% to 40% and $R_{2}^{*}$ values from 20 to 90 s^−1^. Although this range covers PDFF in fatty liver patients and mild iron overload (45 s^−1^ < $R_{2}^{*}$ < 91 s^−1^ from previous work^16^), higher $R_{2}^{*}$ in patients with severe iron might not be robustly estimated using the proposed protocol. Whereas longer TEs with more fat–water phase difference is beneficial for fat–water separation, quantifying higher $R_{2}^{*}$ requires more echoes placed at shorter TEs. For these cases, the Monte Carlo simulation approach used in this work can be extended to include more relevant parameters and help design dedicated acquisition protocols.

In a previous multi‐center multi‐vendor PDFF phantom study,^45^ the slope of the regression line is in the range of 0.86 to 1.02 at 1.5 T and 0.91 to 1.01 at 3 T using vendor protocols. The intercept of the regression line was in the range of −0.65% to 0.18% at 1.5 T and −0.78% to −0.21% at 3 T. In our phantom experiment at 0.55 T, the slopes of the regression line were 0.956 and 0.949 after RLLR and RMT denoising, demonstrating similar PDFF linearity to that at 1.5 and 3 T. The intercepts of the regression line were 2.06% and 2.02% after denoising. Even though RLLR and RMT denoising can effectively reduce the noise, a higher positive bias in PDFF is still observed at 0.55 T compared with results from 1.5 and 3 T.

From the Monte Carlo simulation and the pelvis experiment, we observed that PDFF and $R_{2}^{*}$ accuracy can be limited when the SNR is too low. This is consistent with previous findings that noise effect can lead to PDFF bias.^18^, ^32^ It is also known that noise can bias $R_{2}^{*}$ estimation if the signal in later echoes is close to the noise floor.^46^ Whereas denoising generally improves precision without impacting accuracy, denoising may reduce quantification bias in cases when original image SNR is relatively low. In the pelvis experiment, we found the denoising performance of RLLR and RMT is dependent on the original image SNR, which is affected by different factors including tissue types, acquisition parameters, proximity to the coils, and g‐factor distribution if PI is used. Therefore, future improvements such as better surface coils with more elements or sampling patterns with reduced g‐factor penalty are also important to further improve PDFF and $R_{2}^{*}$ quantification at 0.55 T. We also found PDFF and $R_{2}^{*}$ had different sensitivities to noise. Without denoising, PDFF in liver ROIs showed larger bias. On the other hand, the mean $R_{2}^{*}$ values in liver ROIs were rather consistent with or without denoising. Nevertheless, both denoising methods can provide less noisy $R_{2}^{*}$ maps for more precise measurements and better diagnostic quality.

The computational bottlenecks for both denoising methods were the calculations of singular value decomposition. In our 3D liver dataset, the average computational time for the denoising step (excluding PI reconstruction and signal fitting) was 1 min 10 s for RLLR denoising and 3 min 30 s for RMT denoising, using a MATLAB script with MATLAB Parallel Computing Toolbox (R2023a, MathWorks, Natick, MA) running on a 64‐Core 2.7 GHz CPU (AMD Ryzen Threadripper PRO 5995WX, Santa Clara, CA). RMT denoising had longer computational time because the singular value decomposition was applied on larger 2D matrices. For both methods, computational time can be further reduced by optimizing the software implementation and running on high‐performance hardware.

Both RLLR and RMT denoising rely on two assumptions: (1) the underlying noise is Gaussian‐distributed, and (2) the low‐rank property exists in local image patches. These requirements are typically met in multi‐contrast MR images after noise statistics are carefully corrected. Therefore, both denoising methods can be potentially applied in many other lower‐field or higher‐field MRI applications in which low SNR is a problem. Even though the noise variance can be objectively estimated using these two methods, the choice of patch size is dependent on the effective signal rank and is usually based on empirical results, as used in this work and in previous locally low‐rank denoising works.^21^, ^25^, ^26^, ^27^ One might need to adjust the patch size for optimal denoising performance for different datasets.

We achieved promising RMT denoising performance in our dataset by using both echo and coil dimensions to construct low‐rank patches. However, this approach can be dependent on the coil configuration and should be used with caution in different datasets or scan setups. For example, if there are limited overlapping coil sensitivities in local regions, the constructed local patches may contain noise‐only columns. The desired signal components therefore have a higher risk of being “drowned” in the sea of noise‐related singular value components.^47^ This can result in losing the desired signal components and lead to errors in final quantitative maps.

Deep learning–based methods are another promising approach for noise suppression.^48^ Many deep learning–based denoising methods for lower‐field MRI rely on supervised learning that requires a database of training data.^49^, ^50^ However, obtaining high‐SNR reference training data from multiple scan repetitions may be difficult for abdominal scans due to breath‐holding requirements. For these cases, the denoising methods investigated in this work can be used to generate training data. Another approach to improve the inherent SNR is to use non‐Cartesian MRI sequences, such as radial MRI, with free‐breathing acquisitions.^51^, ^52^, ^53^ These techniques usually require longer scan times but can also provide robust PDFF and $R_{2}^{*}$ quantification. Because of the higher inherent SNR, there can be more flexibility in the choice of acquisition parameters. A suitable scan protocol using non‐Cartesian sequences can also be designed with the Monte Carlo simulation used in this work.

This study has limitations. First, our studied cohort had a limited size, and none of the subjects had liver iron overload ($T_{2}^{*}$ > 45 ms at 0.55 T^16^). Scans in subjects with high liver iron content should be conducted in future works, and the denoising performance should be further validated. Second, our phantom analysis only included fat‐only and $R_{2}^{*}$‐only vials, which may not reflect the actual in vivo environments in the liver, where both fat and iron may be present, although this condition is rare. Further experiments should be done in phantoms with different combinations of PDFF, $R_{2}^{*}$, and T_1_ values^54^ to investigate the denoising performance and the limitations.

## 5 CONCLUSION

We used a Monte Carlo simulation to design an acquisition protocol for PDFF and $R_{2}^{*}$ quantification at 0.55 T with validation in phantom experiments. We showed that both RLLR and RMT denoising improved quantification accuracy in terms of closer agreement with the reference, and improved quantification precision in terms of reduced SDs across scan repetitions. In a cohort with healthy volunteers and fatty liver subjects, RLLR and RMT denoising both improved quantitative maps in terms of the significant decrease of PDFF and $R_{2}^{*}$ SDs in liver ROIs when compared with conventional reconstruction.

## FUNDING INFORMATION

Funding support was provided by the National Science Foundation (NSF) grant 1828736 and the National Institutes of Health (NIH) grants R01DK124417 and U01EB031894.

## CONFLICT OF INTEREST STATEMENT

Coauthor Sophia X. Cui is an employee of Siemens Healthcare.

## Supporting information


**Figure S1.** Monte Carlo simulation results regarding the accuracy and precision for PDFF and $R_{2}^{*}$ mapping using different flip angles (FA), first echo time (TE), and ∆TE at 0.55 T. To balance between accuracy and precision of parameter quantification and breath‐holding scan time, we chose first FA = 8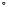, TE = 2.16 ms, and ∆TE = 2.16 ms as indicated by the stars.
**Figure S2.** Comparison of (a) coil‐combined echo 3 (out‐of‐phase) and (b) coil‐combined echo 6 (in‐phase) images from different reconstruction methods All the images are displayed using the same window/level.

## Data Availability

The MATLAB code with some example datasets is available from: https://github.com/HoldenWuLab/LLR‐image‐denoising
