# Supplementary material for: Improved liver fat and R2* quantification at 0.55 T using locally low‐rank denoising
Source: Magn Reson Med. 2024 Oct 9;93(3):1348–64. doi: 10.1002/mrm.30324 (PMC11680733; doi:10.1002/mrm.30324)
Supplement: Supplementary file 1 — Figure S1. Monte Carlo simulation results regarding the accuracy and precision for PDFF and R2* mapping using different flip angles (FA), first echo time (TE), and ∆TE at 0.55 T. To balance between accuracy and precision of parameter quantification and breath‐holding scan time, we chose first FA = 8, TE = 2.16 ms, and ∆TE = 2.16 ms as indicated by the stars. Figure S2. Comparison of (a) coil‐combined echo 3 (out‐of‐phase) and (b) coil‐combined echo 6 (in‐phase) images from different reconstruction methods All the images are displayed using the same window/level. [file MRM-93-1348-s001.docx]

**SUPPORTING INFORMATION**


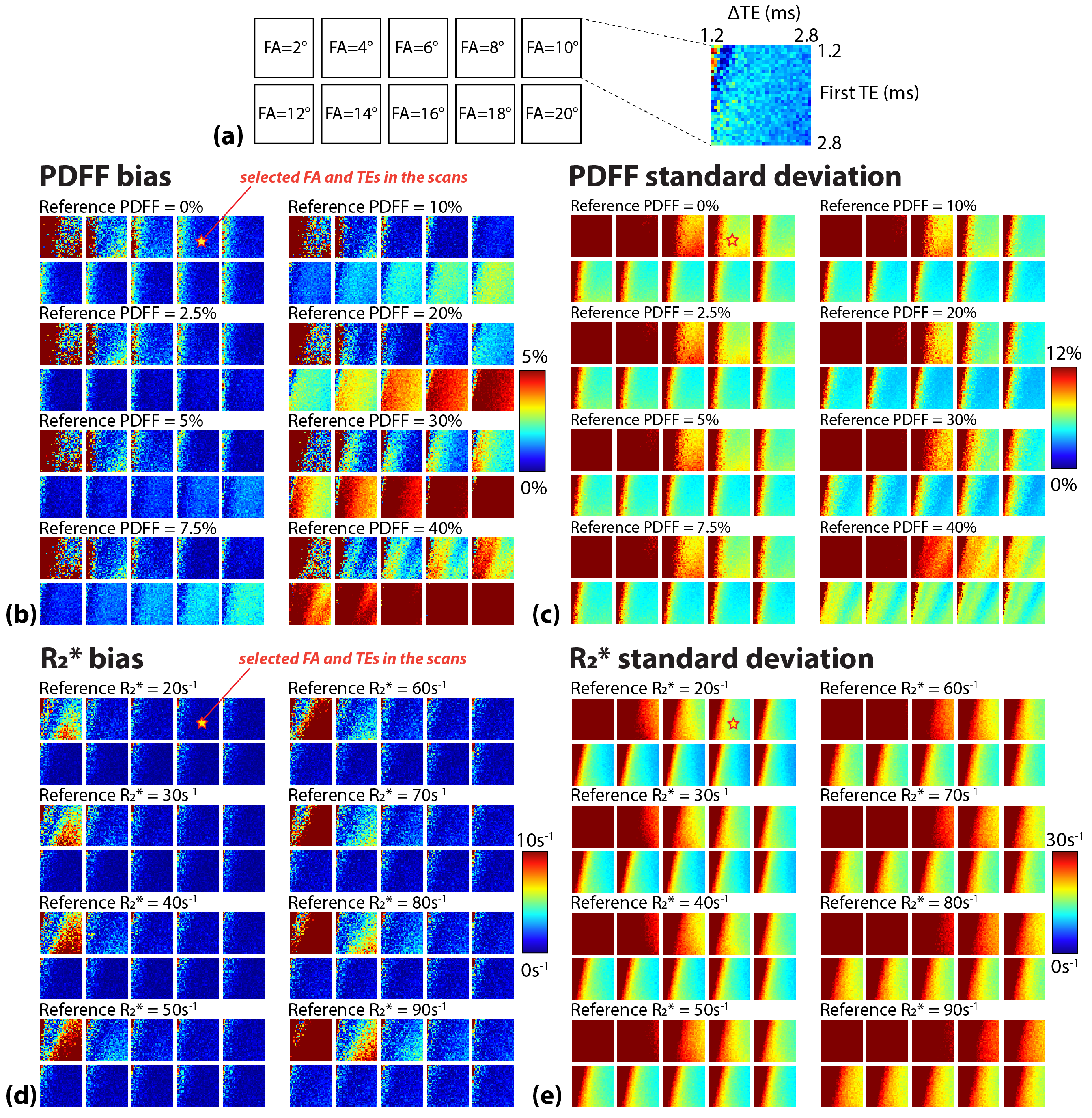


**Supporting Information Figure S1**. Monte Carlo simulation results regarding the accuracy and precision for PDFF and R_2_* mapping using different flip angles (FA), first echo time (TE), and $\Delta$TE at 0.55 T. To balance between accuracy and precision of parameter quantification and breath-holding scan time, we chose first FA=8$^{\circ}$, TE=2.16 ms, and $\Delta$TE=2.16 ms as indicated by the stars.


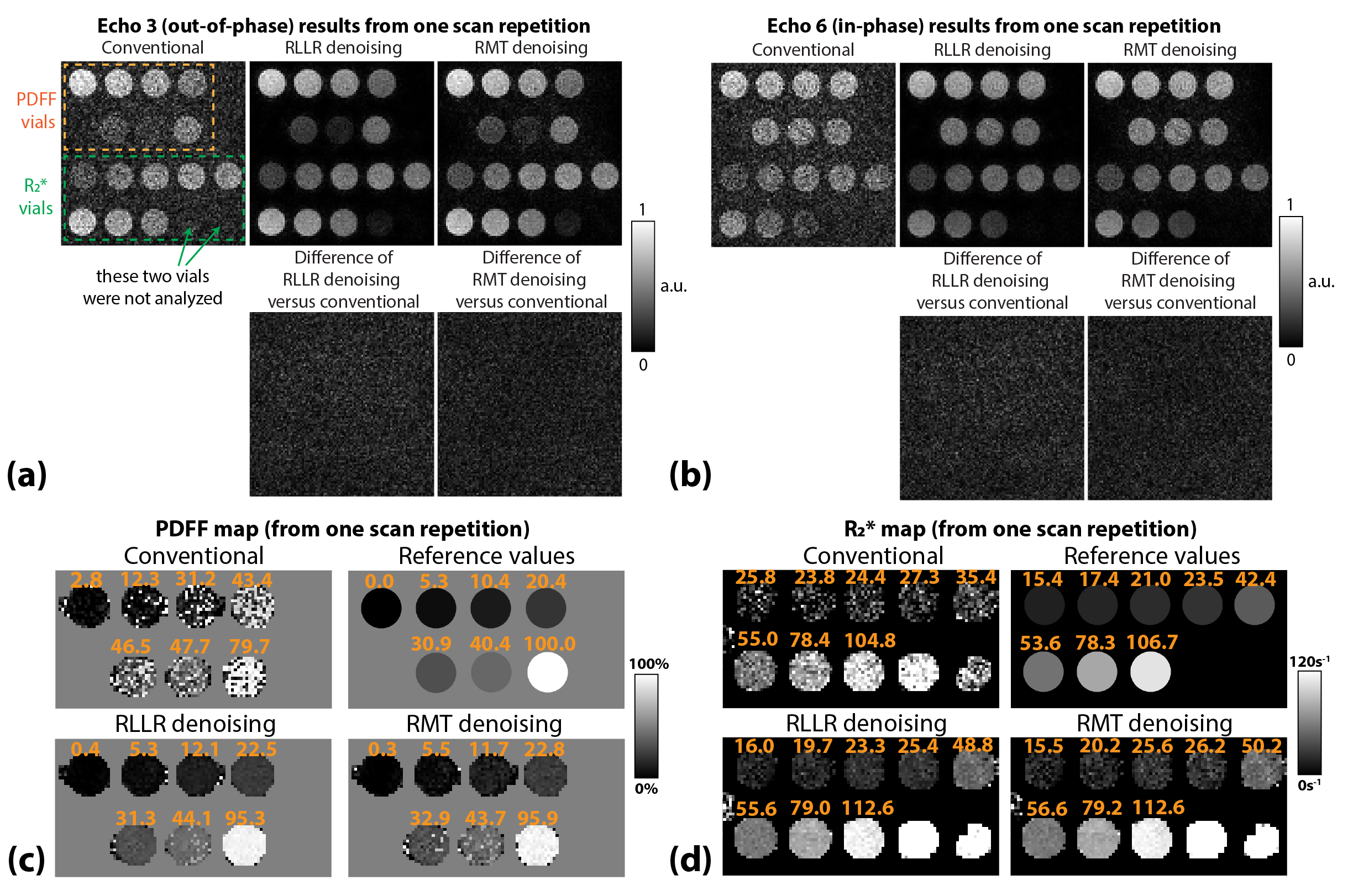


**Supporting Information Figure S2**. Comparison of **(a)** coil-combined echo 3 (out-of-phase) and **(b)** coil-combined echo 6 (in-phase) images from different reconstruction methods All the images are displayed using the same window/level.
